# A Bridge Role Metric Model for Nodes in Software Networks

**DOI:** 10.1371/journal.pone.0111613

**Published:** 2014-11-03

**Authors:** Bo Li, Yanli Feng, Shiyu Ge, Dashe Li

**Affiliations:** 1 Key Laboratory of Intelligent Information Processing, Shan Dong Institute of Business and Technology, YanTai, Shandong, China; 2 Department of Computer Foundation Studies, Shan Dong Institute of Business and Technology, YanTai, Shandong, China; UMIT, Austria

## Abstract

A bridge role metric model is put forward in this paper. Compared with previous metric models, our solution of a large-scale object-oriented software system as a complex network is inherently more realistic. To acquire nodes and links in an undirected network, a new model that presents the crucial connectivity of a module or the hub instead of only centrality as in previous metric models is presented. Two previous metric models are described for comparison. In addition, it is obvious that the fitting curve between the 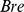 results and degrees can well be fitted by a power law. The model represents many realistic characteristics of actual software structures, and a hydropower simulation system is taken as an example. This paper makes additional contributions to an accurate understanding of module design of software systems and is expected to be beneficial to software engineering practices.

## Introduction

Large-scale software systems have developed quickly with the rapid development of software engineering. Hence, understanding, measuring, and controlling design are significant challenges for designers, which have attracted a significant amount of attention. There are many studies on software metric methods such as property-based [1],junction point [2],productivity [3] and combination [4], but “a common approach of using simple regression models to predict software defects [5]–[8] can lead to risk management decisions”. In 2002, complex networks were applied to metric software structures by Valverde et al, where the software structure is represented by a complex network. Characteristics of scale-free and small-world networks have been determined [9], and subsequently, studies have also determined that software networks that are extracted from various software also follow power-law degree distributions [10]–[19], exhibit strong community phenomenon [10], [20], and show some complex network behavior characteristics[13], [21]–[26]. Furthermore, other studies have been analyzed in software systems, and subsequently, the methodology of dependability in software networks based on three dimensions of structure has been discussed, and the structural stability in software is analyzed on dimension of composition. Because of the reusability of design patterns in object-oriented software systems, the design patterns are regarded as a typical structure that has more effect on the whole [27], [28]. [29] has studied nine large object-oriented software networks, recovering that graphs associated with these software networks are self-similar. They have also studied the time evolution of fractal dimensions during software system growth, and a significant correlation is found between the complexity metrics and the fractal dimension. [30] has presented a systematic empirical analysis of the statistical properties of communities.

On the other research front, some studies are trying to develop a metric for the role of a module in software networks, but few models can describe the “bridge” role of a module more accurately. The Weighted OO Software Coupling Network as the node weight is proposed in [31], where the weight and out degree follow a power law distribution. [32], [33] have introduced main metric parameters of software networks in detail and have integrated these metrics parameters into a hierarchical metric set. The analytic results in [34] have revealed that most of the parameters in complex systems can also be used to represent properties of software structures, some efficient metrics and methods are introduced which are based on basic parameters in other complex systems, and a practical example is used to demonstrate the validity and effectiveness of the proposed metrics. [35] has described some recent algorithms that appear to work as well as some algorithms based on betweenness, which is one of the most important metrics of the centrality of a module in a software network. [36] has introduced another important metric model: closeness. It makes regular and macroscopic analysis and subsequently, utilizes the method to measure important features and characteristics. The relativity among the integral measure and identities facilitates important proofs for the qualification of software qualities.

This paper is motivated by the above considerations. [10], [32]–[33] have proposed some metric parameters and models to represent properties of software structure that are independent of the connectivity role of a module, and modules in [34]–[36] represent the centrality of a module in software networks but are different from connectivity. Hence, a new model is proposed from a new perspective. Some modules behave stronger connectivity than other modules, and if a fault occurs, neighbors of these modules cannot connect to each other. A “bridge” is used to represent the connectivity of the module in the software network; therefore, a bridge role metric model that can more accurately serve as a metric for characteristics is proposed. The remainder of this paper is outlined as follows. After describing the bridge role metric model in section 2, we compare this model with two other previous models and analyze the correlation between the 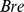 results and other fundamental metrics. In section 4, an actual hydropower system is taken as an example to demonstrate the validity of the model and the implications of design principles for software structure are discussed. In section 5, the conclusion is presented and future studies are proposed.

## Methods

### Software networks

**Figure 1:**
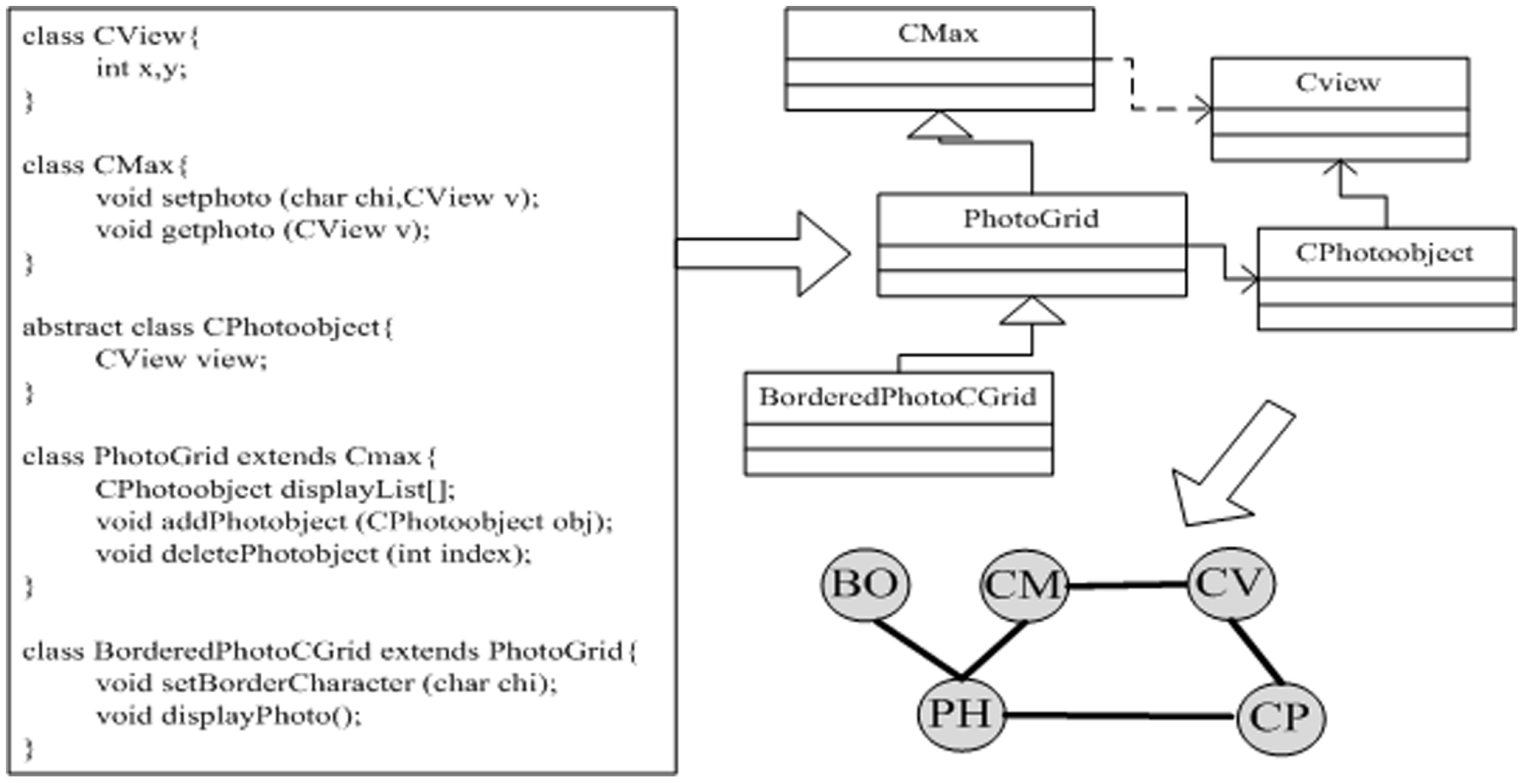
The extraction from codes to software network. The process is as follows: The UML class diagram is first abstracted from the source code and subsequently converted to the undirected software network.

Software is a system which is composed of many interactional and collaborative units reflecting coding, design and execution. The extraction from codes to network is displayed in Fig. 1. Particularly, some modules are reused or rely on other modules, and the dependency relations between two modules A and B include two types: inheritance and association. If A makes reference to B (either through association or inheritance) in its definition, there is an edge directed from A to B and vice versa. Hence, a software network is defined as 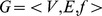, where 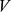 = 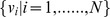, which represents modules, is a set of nodes and *E* = 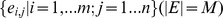, which denotes relations between modules. The repeated edges between modules are not considered. Software is regarded as an undirected network, and 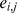 = 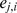. Node *i* is characterized by parameters such as the degree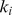, closeness 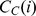, and the betweenness 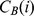, which are presented in section 3. In this paper, approximately 100 randomly selected software (listed in table in Appendix S1) from the open source community (http://sourceforge.net, http://code.google.com/hosting/ and http://www.oschina.net) are chosen as empirical cases.

### Bridge role metric model

As mentioned above, two metric models can better measure the centrality of a node in the software network, *i.e.* the closeness 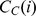 [36] and the betweenness 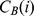 [35], and their definitions are as follows: 
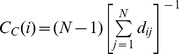
, where *N* is the number of nodes in the software network, *i*<>*j*, and 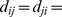
1 if there is an direct connection between node *i* and *j*; otherwise, 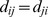 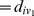
+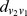
+……+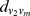
+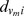, *m*<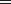 *n*. 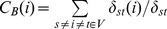, where 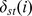 = 1 if node *i* is located on the shortest path between node *s* and *t*, and 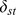 is the number of shortest paths between *s* and *t*.

Conversely, closeness and betweenness cannot show the connectivity role effectively; therefore, the bridge role metric model is proposed in this paper, and comparisons with the two metric models above are executed.

The definition is as follows: 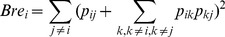
(1)

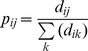
(2)where node *j* is any neighbor of node *i*, and 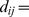
1 if there exists an edge between node *i* and node *j*; otherwise, 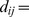
0. We now discuss the value of equation (1). We suppose the number of all neighbors of node *i* is *n* (1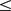 *n* 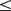 *N-1*,); therefore, node *i* and all its neighbors can be considered as a community as follows: 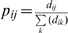 and 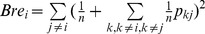. We set the number of neighbors (including node *i*) of node k 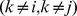 as 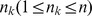 and the number of neighbors of node *j* as 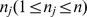. We obtain
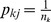
and 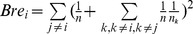. One of the two extreme cases is that when 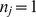
(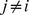),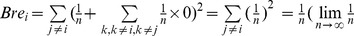 = 0), and the other extreme case is that when 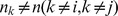, *i.e*., the community is a *n*+1-clique, then 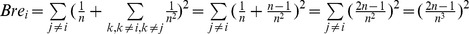. We set *Y* = 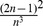, and subsequently, the solutions of equation *Y*′ = 0 are *n* = 0.5 and *n* = 1.5, but *n* is an integer; hence, the extremal solution is *n = 2* and 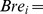
1.125. When 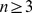,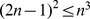; therefore, *Y*<1. Finally, another case should be discussed in which the community is not a star network or a clique. In these cases, the maximum can be computed with the recurrence method. Suppose that there are 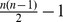 edges between neighbors of node 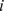, *i.e. M* = 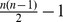
(if *M* = 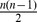, the community is an 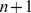-clique). We then set 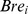
(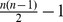) to represent the value of the metric model. We obtain 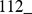
(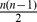) = 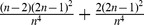; hence, we have 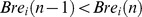 and



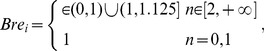



where 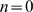 means that the node is an isolated one.

Computing the 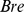 value can be described with the following algorithm. The method of creating the hierarchical network is by placing the nodes to a corresponding hierarchy based on its centralization. For example, the nodes that are in the center will be placed in the most inner, and the whole network will be similar to a multi-ring network.

**Algorithm 1** 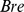
(*N*,*M*)

Create Hierarchical Network *H*(*N*,*M*)

If the number of nodes in the nodes set |*N*|
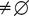


Computing the isolated and nodes (suppose the number of these nodes is 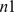,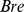 = 1)

hierarchy  = 1

  while |*N*|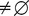 do

   |*N*| = |*N*|-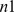;

   Computing the nodes of the current hierarchy

   hierarchy  =  hierarchy+1;

     Removing the nodes of the current hierarchy from |*N*|;

     end while

     returning results

## Results and Discussion

### Comparisons

**Figure 2:**
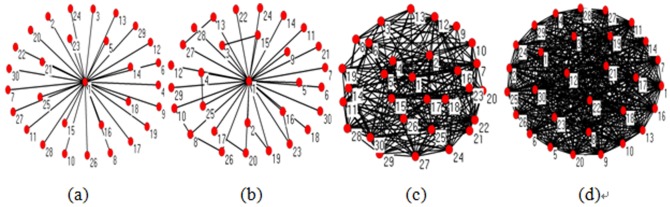
Several cases with number of edges gradually increasing and the fixed nodes. In Figure 2 (a), *N* = 30, *M* = 29, and 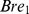 = 0.0345. In Figure 2 (b), *N* = 30, *M* = 37, and 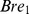 = 0.0577. In Figure 2 (c), *N* = 30, *M* = 275, and 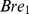 = 0.1319. In Figure 2 (d), *N* = 30, *M* = 435, and 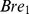 = 0.1332. The value of 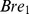 reflects the connectivity of the node 1.

How does the bridge role metric model represent the connectivity of a node? Fig. 2 shows several cases with the number of edges gradually increasing, and node *1* is taken as an example to explain the function of the model. In Fig. 2(a), there are no edges between neighbors of node 1; hence, the neighbors cannot make contact with each other without node 1. In Fig. 2(b), four pair of nodes can connect to one another without node 1, and in Fig. 2(c), much more of these types of nodes exist. In Fig. 2(d), any node can connect to any other one. It can be concluded that the connectivity of a node in a given community becomes stronger with 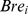 value decreasing, and it has been theoretically proved in section 2.2 with equation (3), where connectivity means the ability to make other nodes communicate with each other.

As mentioned in section 2.2, two previous metric model parameters are the closeness 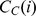 [36] and the betweenness 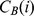 [35]. How does the metric model in this paper work more effectively than these two models? Node 1 is taken as an example, and comparisons are described as follows.

**Figure 3:**
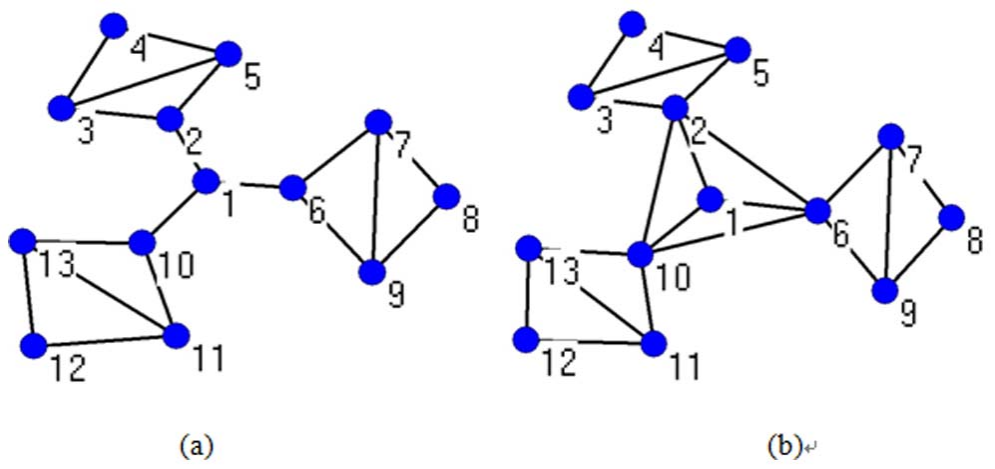
Comparisons of the value between closeness and bridge role in two identical networks. (a) *N* = 13 and *M* = 18. (b) *N* = 13 and *M* = 21, where there are three more edges in this network on the basis of the network in (a). Node 1 is in the center in these two networks.

In Fig.3, there are two networks that almost have the same structure except for a few edges. Node 1 is in the center of (a) and (b). In (a), nodes 2, 6, and 10 can communicate with each other only through node 1; hence, node 1 acts as a “bridge”. In (b), all other nodes can connect to each other without node 1; therefore, in the latter network, the role of node 1 is not very important. The closeness of node 1 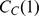 is 0.5 in the two networks, which cannot reflect the evident different role of node 1 as an intermediate node. Nevertheless, in the former network, 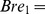
0.3333, and 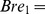
0.6533 in the latter network. It shows that the bridge role metric model can reflect the connectivity of a node more effectively than can the closeness.

**Figure 4:**
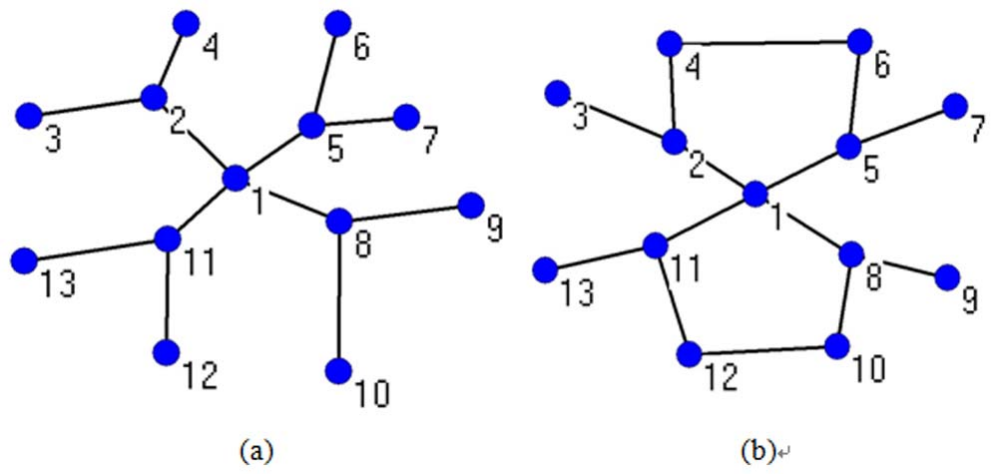
Comparisons of the value between the betweenness and bridge role in two identical networks. (a) *N* = 13 and *M* = 12. (b) *N* = 13 and *M* = 16. Node 1 is also in the center in these two networks as shown in Figure 3.

We now concentrate on the other previous metric model parameter: betweenness. In addition, there are two networks in Fig. 4, where the latter one has two more edges than the former. In (a), there are a total of 66 shortest paths between the nodes excluding node 1, in which node 1 is located in 54 paths; therefore, 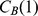 = 0.8182. Meanwhile, node 1 acts as a bridge to allow nodes 2, 6, and 10 to communicate with each other, where 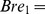
0.25. In (b), the connectivity of node 1 for nodes 2, 6, and 10 does not change, and 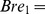
0.25; however, the shortest paths that node 1 is located in decrease, where 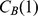 = 0.6667. It should be noted that 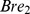,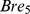,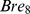 and 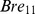 are altered because of the two extra edges.

The conclusion can be drawn as discussed above that the metric model proposed in this paper can reflect the connectivity of nodes more effectively than the closeness or betweenness.

### Simulations

**Figure 5:**
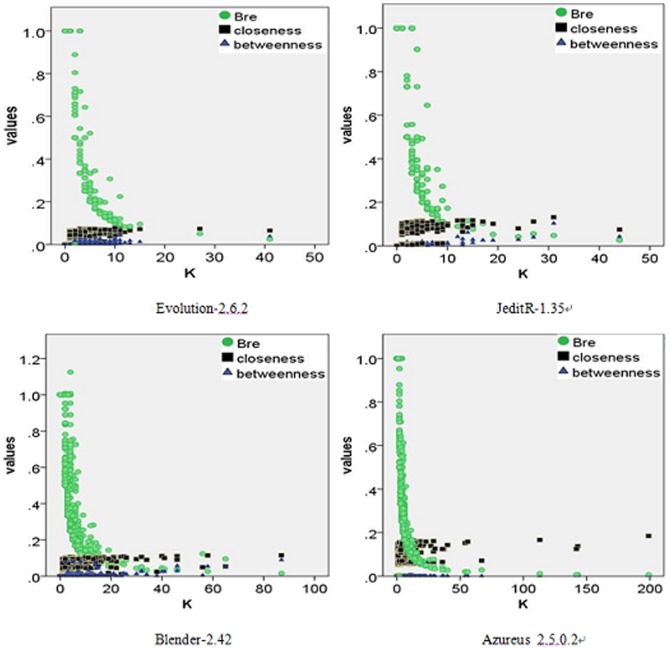
The correlations between the metric model *Bre* values, closeness, betweenness and the degree *K*. The corresponding data obtained from Evolution-2.6.2 (*N* = 1445, *M* = 1129), JeditR-1.35 (*N* = 822, *M* = 718), Blender-2.42 (*N* = 2426, *M* = 2848) and Azureus_2.5.0.2 (*N* = 2375, *M* = 3278), which are well-known software packages. The data points (•,▪,▴) represent measurements of the three metric models.

Some studies have revealed that software networks follow power law distributions over an extent of degree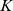, which is the number of edges attached to the node 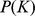
∼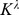 [36]. It is natural to consider the correlations between the bridge role metric model and other metrics. Fig.5 shows the correlations of 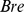, betweenness, closeness, and the degree 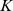 in four familiar software networks.

Typically, centrality (closeness or betweenness) has a significant correlation with the degree; nevertheless, it can be seen in Fig. 5 that the closeness or betweenness increases but is less pronounced as the degree *K* increases, and the centrality of a node does not significantly depend on its degree. Specially, it is determined that 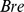 values are logarithmic with the degrees, and it indicates that the node plays a less important connectivity role with increasing degree. Meanwhile, there are more edges between the neighbors of the corresponding node. The correlation contributes more to an accurate understanding of the module for software engineering practices. If there are some reusable modules in a software system, they will obey the engineering principle where if the reusable rate is high. The corresponding module is often redesigned as several additional modules, the neighbors often use or rely on more than one modules, and hence making the neighbors more “close”.

**Figure 6:**
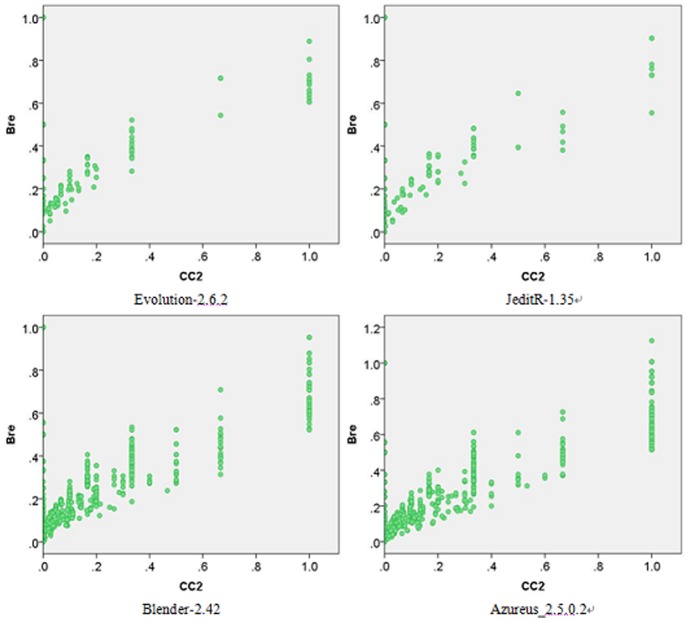
The correlations between the *CC*2 and *Bre* values. The corresponding data are also obtained from Evolution-2.6.2, JeditR-1.35, Blender-2.42 and Azureus_2.5.0.2. The data points • represent measurements of the 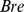 models.

**Figure 7:**
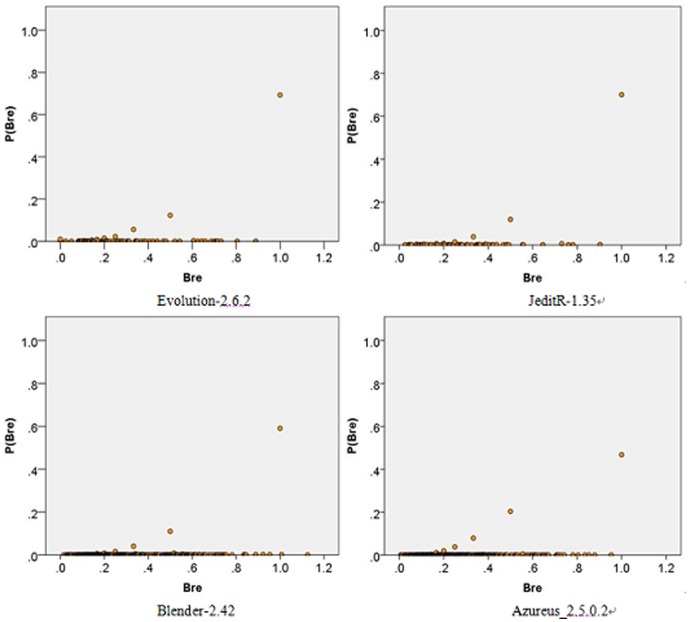
The distributions of the *Bre* values. The data points • represent *P*(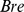). The distribution can be plotted with two branches, one of which seems to be most likely proportional to 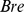 but with few data points; therefore, there is no correlation between 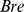 and *P*(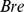).

The 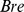 values have a close relation with the edges between their neighbors; therefore, there is most likely a correlation between them and another metric model called Clustering Coefficients (*CC*2) [34], which also depends on the edges. *CC*2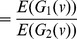, where 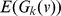 is the number of edges among nodes in the 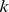-neighborhood of node 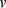
(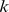 = 1,2). As seen in Fig.6, there is an approximately linear correlation between CC2 and 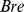. The correlation indicates that increased use between parts of neighbors (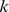 = 1,2) will inevitably lead to a decrease of the connectivity of the corresponding module. Because of the scale-free characteristic (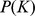
∼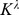) mentioned above, it is clear that 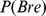
is not proportional to 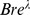, which represents the difference between 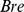 and *K* from another point of view. The 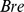
distribution certainly does not reflect the scale-free [26] nature of the software system, which is shown in Fig. 7.

**Figure 8:**
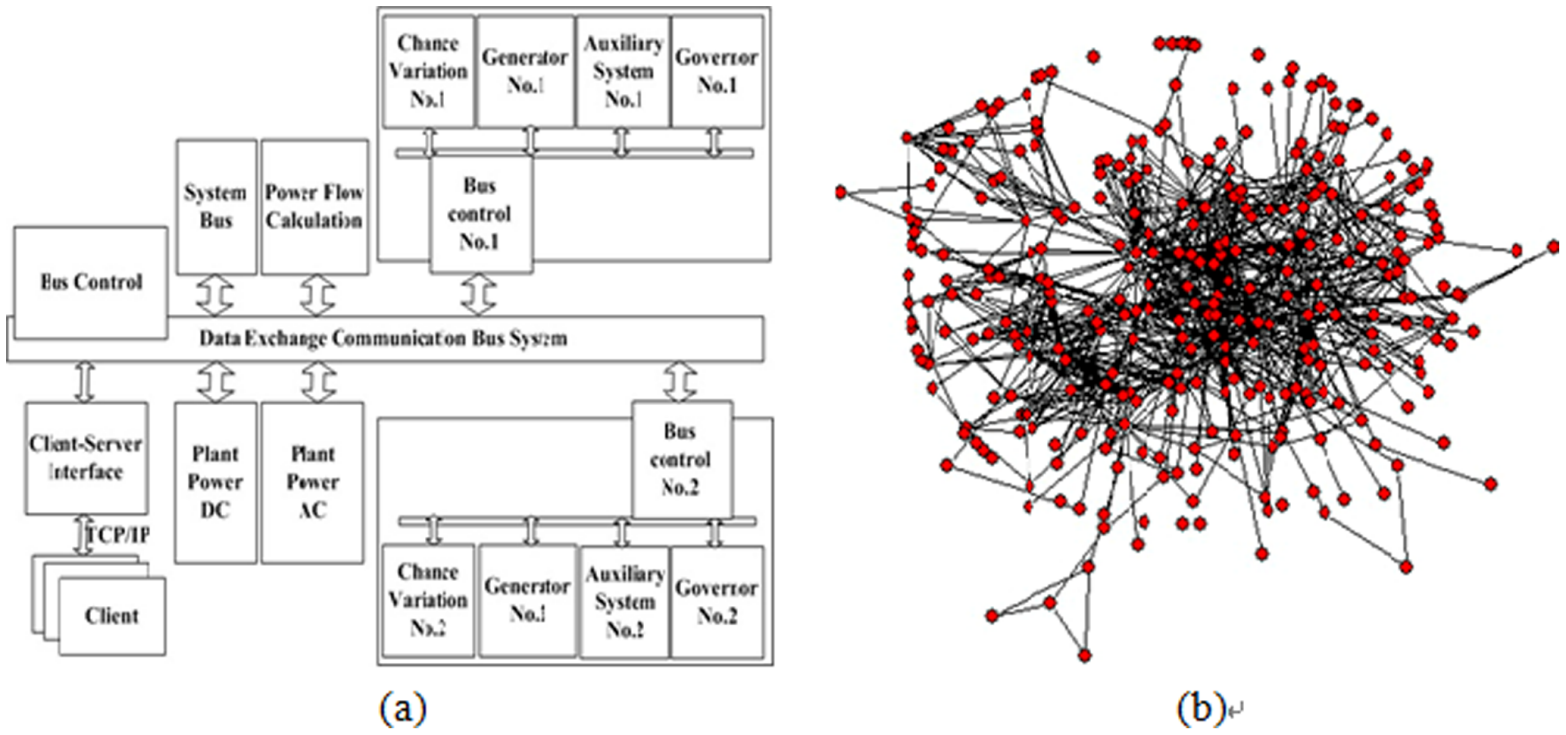
Architecture and network of the hydropower simulation system. (a) Architecture. (b) Network (*N* = 310, *M* = 850). The software system is developed by Embedded Technology Key Lab in Northeastern University (www.netology.cn) for the Fengman hydropower station in China.

To verify the validity of the metric model proposed in this paper for software engineering practices, a hydropower simulation system [34] is taken as an example. The architecture and corresponding networks are shown in Fig. 8. It is developed by Embedded Technology Lab in Northeastern for the Fengman station, which was the earliest established large hydropower station. The software has access to two national software copyright studies (No. 0009448 and No. 050963) and has been working for more than ten years. The metric model in this paper is used for fault detection in developing version 2.0 software. First, modules are sorted based on the 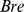 values, subsequently, source codes of the modules that have lower values and are not isolated are analyzed. The studies determined that there are fault-pronesses [37] in four modules (the XJ, RD, LP and VoltCurr modules), which lead to overall instability. These modules are basic control units and plays significant bridge roles in the system because other modules inherit or use them. The studies facilitated redesigns to reduce the fault-proness and enhance stability.

## Conclusions

The contribution of this paper is the proposed bridge role metric model. Because of the different connectivity role of a node in a software network, we use the 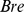 metric model instead of the previous two metric models: betweenness and closeness. After providing a definition, the range of the metric value was discussed. The metric model's function is illustrated with different cases as well as theoretically. Comparisons are also carried out, and the analysis indicates that the model can reflect the connectivity more effectively. Furthermore, it is determined that 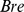 values are logarithmic with the degrees and are proportional to another metric model-Clustering Coefficients-CC2, which indicates that the node plays a less important connectivity role as the degree increases. Nevertheless, 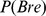 is not proportional to 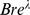. To verify the validity of the model in software engineering practice, a hydropower simulation system is taken as an example to detect the fault-proness in modules.

However, we still require further work to improve the application of the model in software structure designs. Most likely, we can detect fault-proness through a combination of this model and others (*K*, closeness, etc.). Second, it also required some other proof, we will use some software engineering metrics such as coupling, Cohesion to support the solution proposed. Additionally, further investigations to extend the metric model to macro- and micro-structure should be carried out to emphasize estimating the role of a node in the entire software network more effectively.

The work in this paper could facilitate a better understanding of the role of modules in systems. Actually, because local instability most likely leads to global failures, the structure is very important for designers to predict the fault-proneness of a module. The metric can help us to redesign the structure of software, improve the quality of software, and subsequently shorten the development life cycle.

## Supporting Information

Appendix S1 **Appendix to the manuscript.**
(DOC)
