# Supplementary material for: A Bridge Role Metric Model for Nodes in Software Networks
Source: PLoS One. 2014 Nov 3;9(11):e111613. doi: 10.1371/journal.pone.0111613 (PMC4218783; doi:10.1371/journal.pone.0111613)
Supplement: Appendix S1 — Appendix to the manuscript. (DOC) [file pone.0111613.s001.doc]

The related metrics and Attached Table S1 are as follows:

1 **Core**: *k*-core is a subnetwork of software network where each node has at least *k* neighbors in the same core and if node A is in *k*-core but not in *k*+1-core, its core is *k*.

2 **Shortest Paths**: length of the shortest path between 2 nodes

**Table S1.** Related metrics on 100 selected software network

| **Number** | **Software** | **Number**  **of Nodes** | **Number of edges** | **Average Degree** | **Average Core** | **Average**  **Shortest Path** |
| --- | --- | --- | --- | --- | --- | --- |
| 1 | amarok-1.4.1-beta1 | 645 | 665 | 1.3008 | 1.3008 | 3.0437 |
| 2 | aMule-2.1.3 | 639 | 684 | 1.3177 | 1.3177 | 2.9336 |
| 3 | anjuta-2.1.0 | 756 | 557 | 0.9272 | 0.9272 | 2.0268 |
| 4 | apache-tomcat-6.0.18 | 1522 | 2140 | 1.6117 | 1.6117 | 2.3300 |
| 5 | abiword2.2.7 | 2002 | 2978 | 1.2944 | 1.2944 | 1.8700 |
| 6 | asterisk-1.2.9.1 | 465 | 522 | 1.5333 | 1.5333 | 6.6740 |
| 7 | audacious-1.1.1 | 597 | 409 | 0.8610 | 0.8610 | 2.1126 |
| 8 | Azureus_2.5.0.2 | 2375 | 3278 | 1.5996 | 1.5996 | 2.1924 |
| 9 | beagle-2.2.0 | 220 | 288 | 1.4091 | 1.4091 | 2.1043 |
| 10 | bibletime-1.6 | 159 | 162 | 1.2201 | 1.2201 | 1.5856 |
| 11 | binutils-2.17.50.0.16 | 1583 | 1763 | 1.4327 | 1.4327 | 2.4500 |
| 12 | blender-2.42 | 2426 | 2848 | 1.4110 | 1.4110 | 6.5993 |
| 13 | cglib-src-2.0.2 | 1300 | 239 | 1.7931 | 1.7931 | 1.8833 |
| 14 | cinelerra-cvs-20060619 | 2506 | 6391 | 2.7654 | 2.7654 | 5.7575 |
| 15 | clucene-core-0.9.23.0-Source | 320 | 353 | 1.2906 | 1.2906 | 2.6967 |
| 16 | cmake-2.4.6 | 484 | 424 | 1.0186 | 1.0186 | 3.5686 |
| 17 | coda-6.0.14 | 440 | 547 | 1.4614 | 1.4614 | 2.3413 |
| 18 | dasher-4.0.4 | 213 | 302 | 1.6808 | 1.6808 | 2.6660 |
| 19 | dia0.95R | 459 | 679 | 1.6275 | 1.6275 | 2.7254 |
| 20 | dovecot-1.0.rc5 | 371 | 729 | 2.3693 | 2.3693 | 3.7233 |
| 21 | dxr3player-0.10 | 297 | 418 | 1.7172 | 1.7172 | 4.4831 |
| 22 | eclipse3.1.1 | 14739 | 27576 | 2.0742 | 2.0742 | 1.3175 |
| 23 | electric-7.00 | 1445 | 1129 | 2.3350 | 2.3350 | 3.9168 |
| 24 | eMule0.49b | 627 | 580 | 1.1053 | 1.1053 | 2.9168 |
| 25 | epiphany-2.14.2.1 | 320 | 213 | 0.8469 | 0.8469 | 2.6902 |
| 26 | f-spot-0.1.11 | 517 | 537 | 1.2302 | 1.2302 | 1.8813 |
| 27 | fastdb-2.92IDE | 200 | 294 | 1.8000 | 1.8000 | 1.3836 |
| 28 | ffmpeg-0.4.9-p20060302 | 370 | 428 | 1.3649 | 1.3649 | 1.9929 |
| 29 | fontforge-20060703IDE | 522 | 1013 | 2.3640 | 2.3640 | 2.3364 |
| 30 | freemind0.4 | 133 | 102 | 0.9023 | 0.9023 | 2.9350 |
| 31 | freeradius-1.1.0 | 170 | 118 | 1.0235 | 1.0235 | 1.7317 |
| 32 | freetype-2.1.10 | 329 | 374 | 1.3891 | 1.3891 | 2.9614 |
| 33 | gaim-2.0.0beta3 | 490 | 379 | 0.9796 | 0.9796 | 0.4138 |
| 34 | ganglia-3.0.2 | 150 | 156 | 1.3533 | 1.3533 | 2.5385 |
| 35 | gedit-2.14.3 | 206 | 97 | 0.6311 | 0.6311 | 2.2489 |
| 36 | ghc-6.4.2 | 133 | 155 | 1.5188 | 1.5188 | 2.1049 |
| 37 | glib-2.16.5 | 474 | 391 | 1.0295 | 1.0295 | 1.2792 |
| 38 | glibc-2.3.6 | 961 | 609 | 0.8606 | 0.8606 | 2.6936 |
| 39 | gnokii-0.6.12 | 193 | 129 | 0.8394 | 0.8394 | 1.8929 |
| 40 | gnome-media-2.14.2 | 117 | 69 | 0.7863 | 0.7863 | 1.6715 |
| 41 | gnusound-0.7 | 100 | 96 | 1.1300 | 1.1300 | 3.3346 |
| 42 | gnustep-gui-0.10.3 | 543 | 666 | 1.3407 | 1.3407 | 1.4575 |
| 43 | gossip-0.14 | 159 | 100 | 0.7673 | 0.7673 | 1.4257 |
| 44 | graphviz2.6R | 438 | 521 | 1.4703 | 1.4703 | 2.2188 |
| 45 | httpd-2.2.0 | 493 | 627 | 1.5801 | 1.5801 | 3.1369 |
| 46 | j2sdk-1.4.2 | 16039 | 26369 | 1.8610 | 1.8610 | 4.7816 |
| 47 | jakarta-tomcat-5.0.29 | 1452 | 1731 | 1.3643 | 1.3643 | 2.8701 |
| 48 | jdk-1.5.0 | 19173 | 30396 | 1.8060 | 1.8060 | 4.7037 |
| 49 | jEditR1.35 | 822 | 718 | 1.0438 | 1.0438 | 3.0334 |
| 50 | kaffe-1.1.7 | 7849 | 12120 | 1.7546 | 1.7546 | 3.2603 |
| 51 | kdeartwork-3.5.4 | 162 | 105 | 0.8827 | 0.8827 | 1.5756 |
| 52 | kdebase-3.5.3 | 1677 | 1779 | 1.2701 | 1.2701 | 2.3612 |
| 53 | kdebindings-3.5.4 | 2564 | 1811 | 1.4126 | 1.4126 | 3.6102 |
| 54 | kdeedu-3.5.4 | 1010 | 1031 | 2.0416 | 2.0416 | 1.9030 |
| 55 | kdegraphics-3.5.3 | 2014 | 3349 | 3.3257 | 3.3257 | 3.0623 |
| 56 | kdemultimedia-3.5.3 | 1129 | 1077 | 1.9079 | 1.9079 | 2.5487 |
| 57 | kdepim-3.5.3 | 3518 | 4074 | 2.3161 | 2.3161 | 2.7708 |
| 58 | kdesdk-3.5.4 | 819 | 1063 | 1.5360 | 1.5360 | 2.1877 |
| 59 | kdeutils-3.5.4 | 705 | 609 | 1.0695 | 1.0695 | 2.3159 |
| 60 | kdevelop-3.4.0 | 1453 | 1434 | 1.1796 | 1.1796 | 3.0404 |
| 61 | kicad-20060626 IDE | 212 | 300 | 1.6462 | 1.6462 | 1.3020 |
| 62 | kmplayer-0.9.2 | 203 | 284 | 1.5616 | 1.5616 | 3.5389 |
| 63 | koffice-1.5.0 | 4580 | 5892 | 1.4915 | 1.4915 | 2.3534 |
| 64 | kopete-0.12.1 | 1512 | 2009 | 1.5972 | 1.5972 | 1.0418 |
| 65 | linux1.2.13 | 552 | 347 | 0.8170 | 0.8170 | 2.1574 |
| 66 | maildrop-2.0.2 | 107 | 96 | 1.3084 | 1.3084 | 1.7100 |
| 67 | mozilla3.0.11 | 8354 | 13878 | 1.8816 | 1.8816 | 3.0733 |
| 68 | mplayer-20070814 | 1493 | 1209 | 1.0101 | 1.0101 | 2.0288 |
| 69 | mysql-5.0.56 | 3132 | 3837 | 1.3956 | 1.3956 | 6.0744 |
| 70 | octave2.1.73R | 305 | 364 | 1.4328 | 1.4328 | 2.9059 |
| 71 | ooh323c-0.8.4 | 773 | 1381 | 2.0129 | 2.0129 | 3.5951 |
| 72 | OpenIPMI-1.4.14 | 379 | 543 | 1.6306 | 1.6306 | 3.9670 |
| 73 | openldap-2.2.30 | 276 | 384 | 1.7428 | 1.7428 | 1.5290 |
| 74 | openswan-2.4.5 | 368 | 364 | 1.2880 | 1.2880 | 2.7803 |
| 75 | p7zip_4.42 | 531 | 777 | 1.6252 | 1.6252 | 2.1874 |
| 76 | paludis-0.24.6 | 1093 | 1034 | 1.0393 | 1.0393 | 2.2613 |
| 77 | pingus0.7.2 | 450 | 683 | 1.7800 | 1.7800 | 1.9765 |
| 78 | pl-5.6.60 | 791 | 746 | 1.2111 | 1.2111 | 2.1624 |
| 79 | postfix-2.5.5 | 190 | 213 | 1.3263 | 1.3263 | 1.6163 |
| 80 | qcad-2.0.4.0-1 | 312 | 460 | 1.7019 | 1.7019 | 1.8086 |
| 81 | qemu-0.8.1 | 357 | 237 | 0.9048 | 0.9048 | 1.6773 |
| 82 | qtiplot-0.8.2 | 166 | 152 | 1.1265 | 1.1265 | 2.8714 |
| 83 | resin-3.0.22 | 5076 | 7873 | 1.7650 | 1.7650 | 2.5690 |
| 84 | rhythmbox-0.9.6 | 366 | 342 | 1.0820 | 1.0820 | 2.7084 |
| 85 | rpm-4.4.1 | 1260 | 1294 | 1.2365 | 1.2365 | 3.3682 |
| 86 | scim-1.4.7 | 277 | 254 | 1.2058 | 1.2058 | 1.8700 |
| 87 | Silver.1.6 | 346 | 369 | 1.3671 | 1.3671 | 5.5000 |
| 88 | sim-0.9.4 | 786 | 960 | 1.3741 | 1.3741 | 3.0026 |
| 89 | strongswan-2.6.4 | 312 | 265 | 1.1346 | 1.1346 | 2.8284 |
| 90 | subversion-1.5.6 | 1116 | 788 | 0.8405 | 0.8405 | 2.3000 |
| 91 | synaptics-0.14.4 | 208 | 119 | 0.7308 | 0.7308 | 2.2139 |
| 92 | tk8.4.13 | 363 | 491 | 1.7190 | 1.7190 | 2.8085 |
| 93 | ups-3.38 | 246 | 212 | 1.2561 | 1.2561 | 2.3201 |
| 94 | vlc-0.8.5 | 1250 | 1535 | 1.4288 | 1.4288 | 1.9815 |
| 95 | waimea-0.5.0 | 116 | 193 | 1.9483 | 1.9483 | 3.7262 |
| 96 | wine-1.1.5 | 7943 | 7606 | 1.0909 | 1.0909 | 2.6754 |
| 97 | xen-3.0.2 | 1354 | 931 | 0.8516 | 0.8516 | 2.9500 |
| 98 | xorg-server-1.1.1 | 1113 | 1183 | 1.3738 | 1.3738 | 1.5522 |
| 99 | yasm-0.5.0rc1 | 150 | 191 | 1.5000 | 1.5000 | 2.9407 |
| 100 | ZODB3-3.9.0a12 | 549 | 333 | 0.7942 | 0.7942 | 3.6561 |
